# Influence of Nitrosyl Iron Complex with Thiosulfate Ligands on Therapeutically Important Targets Related to Type 2 Diabetes Mellitus

**DOI:** 10.3390/membranes13070615

**Published:** 2023-06-21

**Authors:** Irina I. Faingold, Yuliya V. Soldatova, Darya A. Poletaeva, Elena N. Klimanova, Nataliya A. Sanina

**Affiliations:** 1 Federal Research Center of Problems of Chemical Physics and Medicinal Chemistry, Russian Academy of Sciences, Academician Semenov Avenue 1, Chernogolovka 142432, Russia; soldatovayv@gmail.com (Y.V.S.); enklimanova@mail.ru (E.N.K.); 2 Medicinal Chemistry Research and Education Center, Moscow Region State University, Mytishchy 142432, Russia

**Keywords:** nitric oxide, nitrosyl iron complexes, model membrane, liposomes, membranotropic properties, anti-glycation activity, aldose reductase, type 2 diabetes mellitus

## Abstract

The high prevalence of type 2 diabetes mellitus (T2DM), and the lack of effective therapy, determine the need for new treatment options. The present study is focused on the NO-donors drug class as effective antidiabetic agents. Since numerous biological systems are involved in the pathogenesis and progression of T2DM, the most promising approach to the development of effective drugs for the treatment of T2DM is the search for pharmacologically active compounds that are selective for a number of therapeutic targets for T2DM and its complications: oxidative stress, non-enzymatic protein glycation, polyol pathway. The nitrosyl iron complex with thiosulfate ligands was studied in this work. Binuclear iron nitrosyl complexes are synthetic analogues of [2Fe–2S] centers in the regulatory protein natural reservoirs of NO. Due to their ability to release NO without additional activation under physiological conditions, these compounds are of considerable interest for the development of potential drugs. The present study explores the effects of tetranitrosyl iron complex with thiosulfate ligands (TNIC-ThS) on T2DM and its complications regarding therapeutic targets in vitro, as well as its ability to bind liposomal membrane, inhibit lipid peroxidation (LPO), and non-enzymatic glycation of bovine serum albumin (BSA), as well as aldose reductase, the enzyme that catalyzes the reduction in glucose to sorbitol in the polyol pathway. Using the fluorescent probe method, it has been shown that TNIC-ThS molecules interact with both hydrophilic and hydrophobic regions of model membranes. TNIC-ThS inhibits lipid peroxidation, exhibiting antiradical activity due to releasing NO (IC50 = 21.5 ± 3.7 µM). TNIC-ThS was found to show non-competitive inhibition of aldose reductase with Ki value of 5.25 × 10^−4^ M. In addition, TNIC-ThS was shown to be an effective inhibitor of the process of non-enzymatic protein glycation in vitro (IC50 = 47.4 ± 7.6 µM). Thus, TNIC-ThS may be considered to contribute significantly to the treatment of T2DM and diabetic complications.

## 1. Introduction

Diabetes mellitus is one of the urgent medical and social problems in the world today. According to international statistics, about 573 million people are living with diabetes worldwide and, by 2030, this number will increase to 643 million people [1]. At the same time, type 2 diabetes mellitus (T2DM) accounts for 90–95% of all cases of diabetes [2]. Type 2 diabetes mellitus (non-insulin-dependent diabetes) is a metabolic disease characterized by chronic hyperglycemia that is associated with the damage, dysfunction or even failure of various organs [3]. Numerous experimental and clinical studies suggest that T2DM causes a combination of endothelial dysfunctions [4]. Nitric oxide (NO) is a decisive regulatory molecule with vast metabolic, vascular, and cellular outcomes [5]. The regulation of NO metabolism is especially important in T2DM, as a result of the fact that the activation of NO synthase (NOS) is under insulin control by the protein kinase Akt pathway [6]. An impaired NO production and activity is found in T2DM [7]. Resveratrol is a naturally found polyphenolic compound that is considered a therapeutic agent for the mitigation of diabetic complications. Resveratrol is known to be able to increase NO production through several mechanisms and prevent NO decomposition by reducing oxidative stress [8].

The discovery of nitric oxide’s signaling role in the cardiovascular and nervous systems was the greatest achievement of medical biochemistry in the latter half of the 20th century. NO is synthetized as a byproduct of L-arginine to L-citrulline by a family of NOS. Nitric oxide is known to play important functional roles in a variety of physiological systems, keeping vascular tonus, thrombosis, and neurotransmission in balance [9].

Numerous experimental and clinical studies indicate that persistent hyperglycemia activates the polyol pathway. Depletion in NADPH, an essential cofactor of endothelial NOS, results in NO deficiency [10]. NO is a highly diffusible free radical species and it reacts with a variety of intracellular and extracellular targets. ROS (superoxide anion radical, in particular) is a major driver of NO deficiency that leads to reduced NO signaling in endothelial dysfunction [11]. Endothelial dysfunction plays a key role in the pathogenesis of diabetic vascular disease [12]. In diabetic patients, hyperglycemia stimulates the production of the advanced glycation end products (AGEs) associated with insulin resistance, endothelial dysfunction, and vascular inflammation [13]. Hyperglycemia-induced AGEs are able to reduce NO production and eNOS expression. The current knowledge on the multifaceted roles of NO improve understanding of the mechanisms implicated in the development and complications of T2DM. Thus, NO donors may be considered to contribute significantly to the treatment of T2DM and diabetic complications.

It was shown that NO donor treatment modulates the adverse effects of high glucose in diabetic renal glomeruli in vivo by preventing diabetes-mediated oxidative and nitrostative stress [14]. NO donors can play a role in glucose uptake in insulin-resistant states. Thus, sodium nitroprusside elicit an increase in glucose uptake in cells derived from patients with T2DM [15]. A number of studies have shown that L-arginine can also have a positive effect on glucose levels in T2DM [16]. Metformin (a biguanide derivative) is commonly prescribed as a drug for the management of T2DM. Recent studies revealed that the activation of AMP-activated protein kinase (AMPK) arranged the therapeutic effects of metformin [17]. Activated AMPK could precisely phosphorylate endothelial nitric oxide synthase (eNOS) in endothelial cells and improve endothelial function [18]. It was also shown that metformin metabolism is accompanied by NO release [19].

Metformin is commonly considered as safe and well-tolerated, however, its gastrointestinal side effects include diarrhea, nausea, and vomiting. Metformin overdose is associated with hypoglycemia and lactic acidosis [20]. Finding new effective antidiabetic compounds remains an important task for medicinal chemistry.

**Figure 1:**
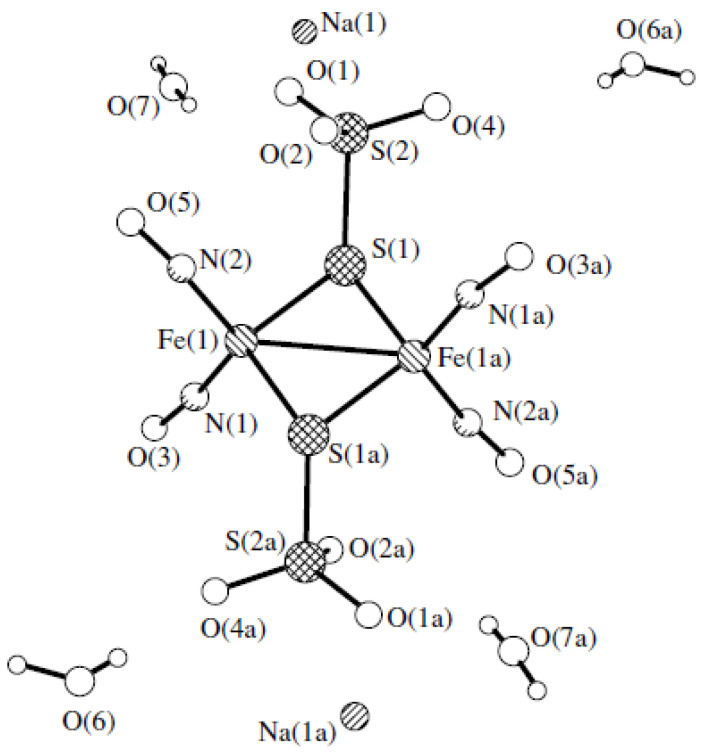
Molecular structure of the nitrosyl iron complex Na_2_[Fe_2_(S_2_O_3_)_2_(NO)_4_]·4H_2_O (TNIC-ThS) (from X-ray data on the work [22]).

Nitrosyl iron complexes are considered to be promising compounds for therapeutic purposes [21]. This work deals with tetranitrosyl iron complex with thiosulfate ligands Na_2_[Fe_2_(S_2_O_3_)_2_(NO)_4_] 4H_2_O (TNIC-ThS) [22] (Figure 1). TNIC-ThS has the ability to prolong NO generation without additional activation in aqueous solutions. It was shown that the IV administration of TNIC-ThS to rabbits led to the formation of protein-bound dinitrosyl iron complexes in plasma [23]. Protein-bound dinitrosyl iron complexes presented in the blood flow for more than two days. After the subsequent insertion of spin trap Fe^3+^–(MGD)_2_, the loss of the essential part of the donor occurs, and the secondary products are freely removed from the blood flow over 1 h [23]. TNIC-ThS is an inhibitor of 3′,5′-cyclic-nucleotide phosphodiesterase, and calcium-magnesium-ATPase of sarcoplasmic reticulum, in the concentration range of 0.001–0.1 mM [24]. The geroprotective effect of TNIC-ThS was also shown; the daily intraperitoneal administration of TNIC-ThS, at a dose of 5 mg/kg, to irradiated Wistar rats restores the level of malondialdehyde and the activity of antioxidant defense enzymes—superoxide dismutase, catalase, glutathione peroxidase and glutathione reductase—and leads to an increase in the life span of rats [25].

The purpose of this work is a comprehensive study of the effect of TNIC-ThS on the therapeutic targets of T2DM in vitro: the ability to bind lipid membranes, inhibit lipid peroxidation (LPO) and non-enzymatic glycation of bovine serum albumin (BSA), as well as aldose reductase, of the enzyme that catalyzes the reduction in glucose to sorbitol in the polyol pathway.

## 2. Materials and Methods

### 2.1. Chemicals and Materials

Phosphatidylcholine from egg yolk (PC) (AppliChem, Darmstadt, Germany), tris (hydroxymethyl) aminomethane hydrochloride (Tris-HCl buffer) (Sigma–Aldrich, St. Louis, MO, USA), sodium phosphate buffer (PBS) (Sigma–Aldrich, USA), trichloroacetic acid (TCA) (Sigma–Aldrich, USA), pyrene (Sigma–Aldrich, USA), eosin Y (Sigma–Aldrich, USA), glucose oxidase (Sigma–Aldrich, USA), catalase (Sigma–Aldrich, USA), bovine serum albumin fraction V (BSA) (Sigma–Aldrich, USA), aminoguanidine-HCl (Sigma–Aldrich, USA), D,L-glyceraldehyde (Sigma–Aldrich, Buchs, Switzerland), D-glucose (Sigma–Aldrich, USA), and NADPH (Applichem, Darmstadt, Germany) were used.

TNIC-ThS (Na_2_[Fe_2_(S_2_O_3_)_2_(NO)_4_] 4H_2_O) was synthesized according to the procedure described in the work [22].

### 2.2. Fluorescent Probes Assay

The interaction of the TNIC-ThS with model PC membranes was assayed by the fluorescent probe method [26], the xanthene dye eosin Y, and pyrene. Liposomes preparation procedure was described in detail in a previous work [27].

Fluorescence studies were carried out using Cary Eclipse fluorescence spectrometer (Varian, Palo Alto, CA, USA). The fluorescence quenching was observed at 530–600 nm for eosin Y and at 350–600 nm for pyrene. All titrations were performed manually with a 1 mL microsyringe. The ratios of pyrene monomer (394 nm) to excimer (475 nm) fluorescence were calculated. The figures of fluorescence quenching spectra were made using Origin 7.5.

The kinetics of eosin Y phosphorescence decay was recorded using Cary Eclipse fluorescence spectrometer (Varian, Palo Alto, CA, USA). Before measurements, oxygen was deleted from the sample by adding 1 μg of glucose oxidase and 1 mg of glucose, as described previously [28].

### 2.3. Tissue Preparation

The work was carried out in accordance with the EU Directive 2010/63/EU and was approved by the Ethical Committee of FRC PCPMC RAS (Approval No 23/1 from 14 February 2023). Hybrid mice BDF1 at the age of 6 months were decapitated. Each liver was quickly removed and frozen in liquid nitrogen. The liver was defrosted and homogenized for 2 min in 0.1 M PBS (pH = 6.2) using a Wisd WiseTis HG-15D homogenizer. The protein concentration in the liver supernatant was determined by the Lowry method [29].

### 2.4. Luminol-Amplified Chemiluminescence Assay

The antioxidant activity of TNIC-ThS was studied by luminol-amplified chemiluminescence during the TBHP-induced oxidation of a mouse brain homogenate [30]. Samples for liquid-phase oxidation were prepared as follows: a solution of luminol (0.5 mM) was prepared by diluting a stock solution of luminol (10 mM) in ethanol (Sigma–Aldrich, St. Louis, MO, USA) with bidistilled water. TBHP solution was prepared by diluting the stock solution (0.73 M) 10 fold with bidistillate. The stock solution of Tris-HCl buffer (0.5 M) was diluted with bidistillate to a concentration of 0.1 M (pH = 7.4). The homogenate was prepared in a Wisd WiseTis HG-15D homogenizer from mouse brain tissue (8 mL of Tris-HCl buffer was taken per 1 g of brain tissue). The mouse brain homogenate was diluted with bidistillate to a protein concentration of 0.1 mg/mL. Luminol luminescence intensity was recorded at 37 °C using Luminometr-1250 LKB Wallak chemiluminometer. The integral intensity of luminol luminescence was calculated using 512 points of the kinetic dependence I = f(t) of the luminol luminescence intensity on the reaction time of the liquid-phase oxidation of the brain homogenate in the presence of TNIC-ThS. The level of free radicals in mouse brain homogenate was assessed by the change in the light sum (the area under the kinetic curve of luminescence intensity).

### 2.5. Aldose Reductase (ALR2) Inhibitory Activity

Supernatant of liver homogenates was obtained using Ohaus frontier 5515R (Parsippany, NJ, USA) centrifuge and then transferred into a new tube. Samples were centrifuged for 30 min at 9000 rpm. The effect of the TNIC-ThS on the catalytic activity of ALR2 was detected spectrophotometrically, using Agilent Cary 60 spectrophotometer (Santa Clara, CA, USA) by change in the absorption of NADPH at λ = 340 nm; results was expressed as decrease in the optical density (O.D.) s^−1^ mg ^−1^ protein [31]. The Ki value and inhibition type of TNIC-ThS were evaluated from the Lineweaver–Burk [32] graphs at TNIC-ThS concentration of 0.2 mM and different concentrations of D,L-glyceraldehyde (substrate).

### 2.6. Antiglycation Assay

Antiglycation activity of TNIC-ThS was assayed by the known method [33], based on the ability of tested complex to inhibit glycoxidation-mediated development of bovine serum albumin (BSA) fluorescence. Fluorescence was measured at an excitation/emission wavelength of 370/440 nm for vesperlysines-like AGE [34]. The concentration which inhibits glycation activity by 50% (IC 50) was determined. As a positive control, the experimental inhibitor of non-enzymatic glycation-aminoguanidine [35] was used.

### 2.7. Acute Toxicity Test Procedure

TNIC-ThS was administered intraperitoneally in graduated doses of 30, 35, 40, 50, 55, and 60 mg/kg to several groups (six animals in each group) of male BDF1 mice weighing 20–22 g according to the method [36]. Sterile physiological saline was used for injections in control group. Mortality and the clinical picture of intoxication were observed for 14 days. LD50 was used as the main toxic characteristic of TNIC-ThS.

### 2.8. Statistical Analysis

All statistical analyses were performed using the statistical software package Origin 9.1 and GraphPad Prism 8. All in vitro assays for the TNIC-ThS were performed in triplicates, values were shown as mean ± standard error of the mean (SEM). Individual groups were compared using the Student’s *t*-test. *p* values < 0.05 were considered statistically significant.

## 3. Results and Discussion

### 3.1. Interaction of TNIC-ThS with PC Liposomes

The interaction of pharmacologically active compounds (PhACs) with membrane lipids plays an important role in predicting pharmacokinetic properties, such as their absorption, distribution, metabolism and excretion, which are essential for the efficacy and safety of drugs [37]. Model membranes are useful for the prediction and simulation of biological membrane processes and for studying membrane–drug interactions under very defined and controlled conditions. Numerous studies have shown a good correlation between the drug–lipid interactions and therapeutic efficacy [38,39,40].

In this work, we studied the interactions of TNIC-ThS with phospholipids, specifically, to assess the level of TNIC-ThS spontaneous insertion into the lipid bilayer. Pyrene is a highly lipophilic probe and can be easily inserted into the hydrocarbon region of lipid bilayer. Formation of excited pyrene dimers (excimers) in liposomal membranes is a diffusion-controlled reaction [41]. The titration of pyrene by TNIC-ThS solution resulted in the efficient quenching of the fluorescence of pyrene, while the excimer to monomer (I′/I) ratio increased. This indicates that the interaction between TNIC-ThS and the excited molecules of the probe is efficient in both monomeric and excimeric states and leads to the complete quenching of excited pyrene molecules.

**Figure 2:**
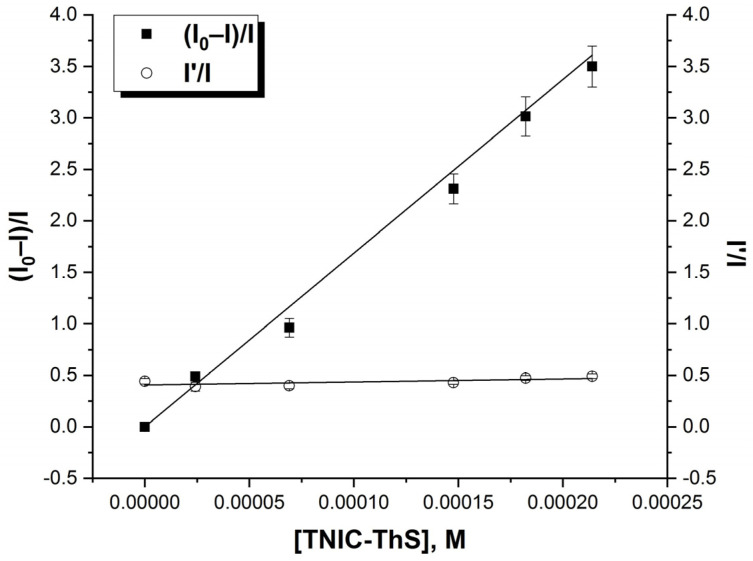
The Stern–Volmer graph for the fluorescence quenching of pyrene as a function of TNIC-ThS concentration and the fluorescence intensity ratio of excimer (I′) over monomer (I) as a function of TNIC-ThS concentration. Liposomes suspension (0.5 mM PC) containing 5.0 μM of pyrene was titrated by consecutive additions of TNIC-ThS solution (1 mM). The excitation and emission slits were adjusted at 5 nm, and the excitation wavelength was 337 nm.

The addition of TNIC-ThS to the liposomes suspension resulted in effective pyrene fluorescence quenching with no changing in the excimer-to-monomer (I′/I) ratio (Figure 2). The Stern–Volmer constant value was K_SV_ = 1.45 × 10^4^ M^−1^. TNIC-ThS did not change the membrane viscosity, as excimer formation requires some freedom of movement of the probe molecules during the lifetime of the excited state and I′/I ratio did not change [42]. This affirms TNIC-ThS molecules’ distribution into the lipid bilayer’s hydrophobic core.

Phosphorescence quenching studies provided a highly sensitive assay for TNIC-ThS interactions with the charged molecules of eosin Y. Eosin Y was shown to be adsorbed on the lipid membrane in the region of the polar headgroups [43]. The effectiveness of phosphorescence quenching, set by values of triplet states quenching rate constants (kq), depends on the character of the triplet label and the quencher, including their charges, the polarity and viscosity of the solvent, and the temperature [44].

**Figure 3:**
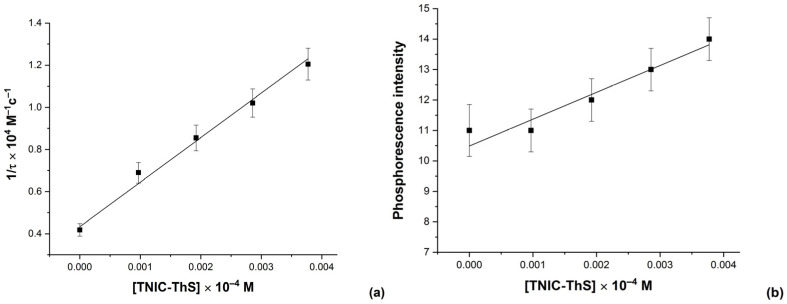
Eosin Y phosphorescence decay rate (**a**) and intensity (**b**) as a function of TNIC-ThS concentration in liposomes suspension. Liposomes suspension (0.5 mM PC) containing 2.0 μM of eosin Y was titrated by consecutive additions of TNIC-ThS solution (1 mM). The excitation and emission slits were adjusted at 5 nm, and the excitation wavelength was 517 nm.

The kinetics of phosphorescence decay indicated the rate of TNIC-ThS molecules interacting with eosin Y (Figure 3). Quenching of the phosphorescence of eosin Y by TNIC-ThS occurs with bimolecular rate constant 2 × 10^10^ M^−l^ s^−1^, which is equal to the diffusion-limited Smoluchowski rate. It was previously shown that, in aqueous solutions, TNIC-ThS decomposes into paramagnetic mononuclear dinitrosyl iron complexes, which gradually decay as well [45]. Thus, the average time an excited eosin Y molecule took to become quenched by TNIC-ThS disintegration products decreased.

This work shows the high ability of TNIC-ThS to bind liposomal membranes. TNIC-ThS molecules interact with membrane lipids in the hydrophobic acyl chain region, close to the glycerol group of lipid molecules, and near the hydrophilic head groups.

### 3.2. TNIC-ThS Radical Scavenging Capacity in Luminol Chemiluminescence Assay

An imbalance between the production and inactivation of ROS by antioxidant defenses leads to oxidative stress, which plays an essential role in the development of diabetes complications, both microvascular and cardiovascular [46]. In biological membranes, the peroxidation of unsaturated fatty acids produces a lot of ROS: hydroperoxides, and carbonyl compounds, which may be cytotoxic [47]. Patients with T2DM mellitus show important evidence of oxidative stress resulting in vascular complications [48]. The increased oxidants in T2DM occur as a result of mitochondrial dysfunction [49] and NADPH oxidase (Nox) [50], induced by hyperglycaemia and dyslipidaemia. Therefore, the development of effective antioxidant therapies is an important goal. Promising therapeutic effects of antioxidants on animal models of T2DM [51,52], as well as in clinical and epidemiological studies [53,54], have been shown. Increased oxidative stress seems to be a harmful factor leading to insulin resistance, dyslipidemia, β-cell dysfunction, and impaired glucose tolerance [54,55,56,57,58,59]. The level of glutathione decreased in patients with T2DM [60] and T2DM is associated with the significant perturbation of the systemic redox state [61].

Previously, in our works [45,62], we have studied the antioxidant activity of TNIC-ThS using levels of malondialdehyde (MDA) and the kinetics of MDA accumulation as markers of LPO in the mouse brain homogenate in vitro. TNIC-ThS effectively reduced the rate of MDA accumulation. A comparison of TNIC-ThS efficacy with NO revealed that the antioxidant activity of the complex appears to be due to the reaction of nitric oxide released upon hydrolysis of TNIC-ThS with lipid radicals.

**Figure 4:**
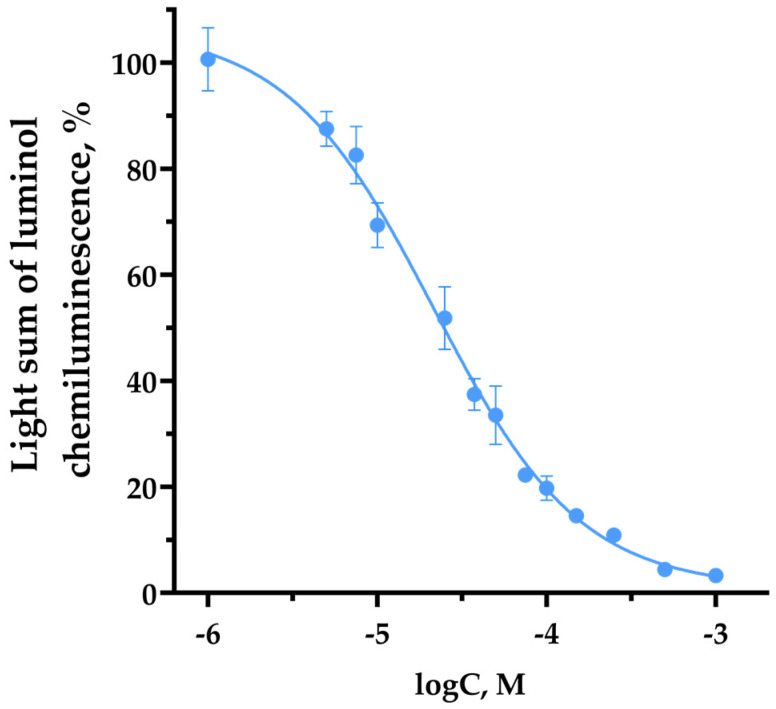
Dose–effect curve of the dependence of the luminol chemiluminescence light sum on the concentration of TNIC-ThS. CL data are shown as a percentage relative to the control. The reaction mixture contained homogenate (protein concentration 0.1 mg/mL), 0.1 M Tris-HCl (pH 7.4), luminol (0.05 mM), TBHP (Sigma–Aldrich, St. Louis, MO, USA) (0.073 M) and TNIC-ThS.

Luminol-amplified chemiluminescence assay is based on the antioxidant-dependent quenching of chemiluminescence [30]. It was observed that TNIC-ThS reduces luminol chemiluminescence intensity in a wide range of concentrations. The dose–response curve is shown in Figure 4. The IC50 value for TNIC-ThS was evaluated as 21.5 ± 3.7 µM. TNIC-ThS is three times more effective than classic antioxidant BHT (IC50 = 70.1 ± 4.0 µM). A probable mechanism of TNIC-ThS antiradical activity is the interaction of released NO with lipid radicals, resulting in the termination of the free radical chain reaction.

Thus, it was shown that TNIC-ThS has a pronounced antioxidant activity and acts as a free radical scavenger.

### 3.3. TNIC Antiglycating Activity

The non-enzymatic glycation of proteins is one of the major pathways involved in the development and progression of diabetic complications such as nephropathy, retinopathy, and neuropathy. Hyperglycemia starts forming covalent adducts with plasma proteins, leading to the formation of advanced glycation end products (AGEs) [63]. The inhibition of AGEs formation has a desirable and beneficial effect on diabetic complications [64]. One of the most studied antiglycating drugs is aminoguanidine, which inhibits AGEs production by blocking reactive carbonyl species, such as methylglyoxal [65,66,67], but it was shown to be toxic at higher concentrations (>10 mM), and the human clinical trials were consequently stopped [68]. Oxidative stress and glycation are closely related [69]. AGEs increase ROS formation and impair antioxidant systems; at the same time, the formation of some AGEs is induced by oxidative reactions. Thus, AGEs promote chronic stress conditions in diabetes [70,71].

Thus, the inhibition of AGEs formation associated with hyperglycemia, as well as the inhibition of LPO, are both targets for therapeutic strategies against a number of pathological events in diabetes [72].

**Figure 5:**
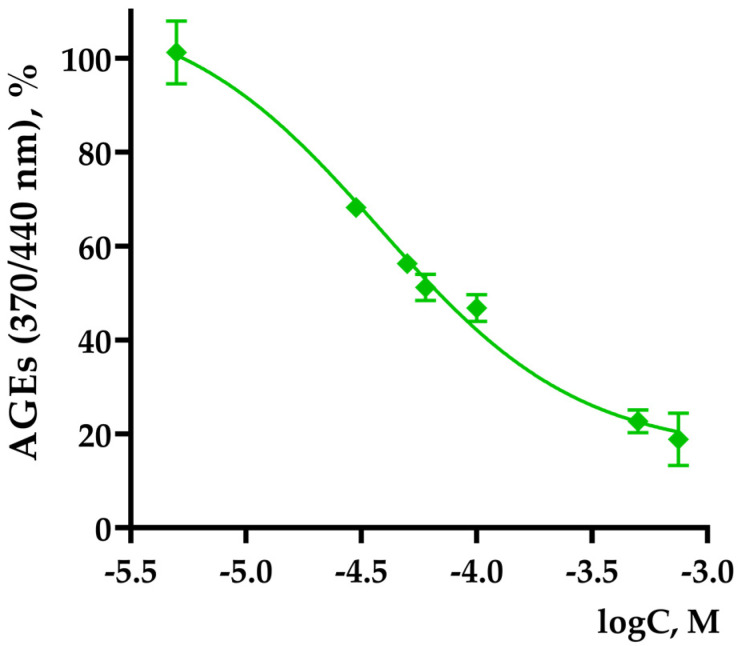
Dose–effect curve for vesperlysines-like AGEs formation in the presence of TNIC-ThS. λexc/em = 370/440 nm. The reaction mixture contained 0.5 mL BSA (4 mg/mL), 0.4 mL D-glucose (0.4 M) in Na-phosphate buffer (pH = 7.4, sodium azide content 0.02%), and 0.1 mL of the TNIC-ThS.

The effect of TNIC-ThS on the BSA non-enzymatic glycation in vitro was studied in this work. TNIC-ThS was found to be an effective inhibitor of glycation. A decrease in AGE fluorescence at wavelengths λexc/em = 370/440 nm, corresponding to the fluorescence of vesperlysines-like AGEs, was found. The dose–response curve is shown in Figure 5.

The IC50 for the formation of vesperlysines-like AGEs was calculated, it was 47.4 ± 7.6 µM. TNIC-ThS is a stronger glycation inhibitor compared to aminoguanidine (IC50 for forming vesperlysines-like AGEs of 1294 μM), which is used in in vitro studies as a positive control.

The antiglycation effect of gaseous NO was estimated. NO dissolved in water inhibits the process of non-enzymatic glycation of BSA by 20% at a concentration of 0.2 mM. A comparison of TNIC-ThS efficacy with gaseous NO indicated that the antiglycation activity of the complex is partially determined by the NO released upon hydrolysis.

The obtained data are consistent with the studies that demonstrated that NO donors and antioxidants are capable of regulating pathways stimulated by AGE. It was shown that nitric oxide donors S-nitroso-N-acetylpenicillamine and sodium nitroprusside, as well as antioxidants N-acetylcysteine and taurine, significantly reduced the synthesis of cGMP, AGE-inhibited NO production, and inducible NO synthase/cGMP-dependent protein kinase activation [73,74,75].

### 3.4. ALR2 Inhibitory Activity

Aldose reductase (ALR2) is the first enzyme of the polyol pathway. ALR2 reduces glucose to sorbitol in an NADPH-dependent reaction. Sorbitol can accumulate in retinal and renal tissues leading to the disruption of cellular homeostasis and development of diabetes-related complications like retinopathy, neuropathy, and nephropathy [76].

It was shown that ALR2 inhibitors can prevent or slow down the progression of pathologies associated with T2DM, like retinopathy, neuropathy, cataracts, and nephropathy, both in animal models and in humans [77]. The polyol pathway is a substantial mechanism whereby high glucose can induce oxidative stress. The reduction in glucose to sorbitol, catalyzed by ALR2, is a rate-limiting step of the polyol pathway. A transgenic mice model, with overexpressed ALR2 in lenses, have shown that glucose flux through the polyol pathway is the major cause of hyperglycemic oxidative stress in this tissue [78]. Increased ALR2 activity contributes to retinal oxidative stress and vascular endothelial growth factor overexpression in early diabetes [79]. ALR2 inhibition neutralizes PARP activation and nitrosative stress in the diabetic renal cortex and in the mesangial cells exposed to high glucose levels [80]. ALR2 inhibition suppresses early apoptosis in the neural retina of diabetic rats [81].

The design and use of ALR2 inhibitors having antioxidant activity is a promising strategic approach that is under active consideration [82,83,84,85]. The multi-target drugs for T2DM treatment is a good strategy as well [67,86].

**Figure 6:**
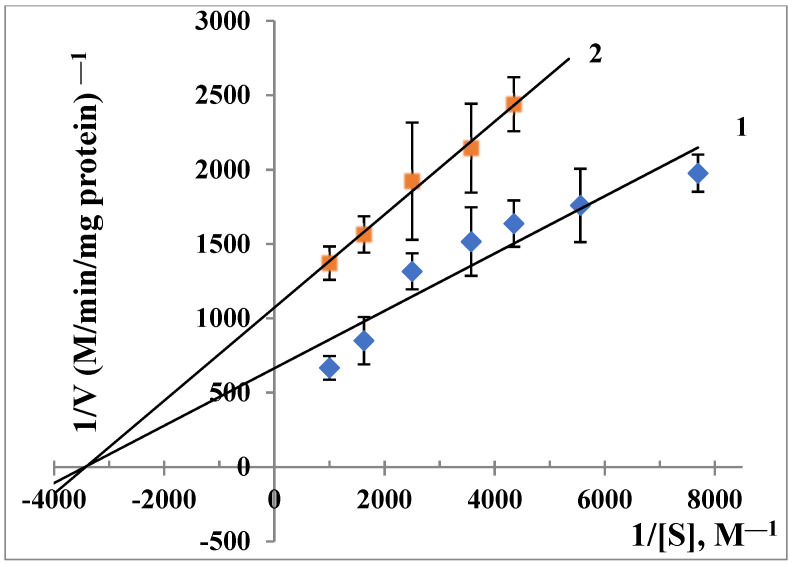
Noncompetitive inhibition of the catalytic activity of ALR2 by 0.2 mM TNIC-ThS. [S]—D,L-glyceraldehyde, V—a rate of changes in NADPH. 1—w/o inhibitor; 2—with inhibitor. The reaction mixture (1 mL) contained 0.3 mL of supernatant, 1 mM D, L-glyceraldehyde, 0.1 mM NADPH in sodium phosphate buffer (pH 6.2), and TNIC-ThS.

In the present study, a supernatant of mice liver homogenate was used as a source of ALR2. It was found that TNIC-ThS inhibits ALR2 by 30 ± 2%. To evaluate the inhibition constants, the Lineweaver–Burk plot was used, displaying kinetic data regarding the reaction rate dependence on the concentrations of substrate in the absence and the presence of an inhibitor. The Ki value was 5.25 × 10^−4^ M. As Figure 6 shows, that type of inhibition was non-competitive. The well-known ALR2 inhibitors used in the clinic—sorbinil, tolrestat, zopolrestat, ponalrestat, and epalrestat—are known to be non-competitive inhibitors [87].

ALR2 is very sensitive to oxidants due to a reactive cysteine (Cys-298) in the active site. NO induces the nitrosation of accessible cysteines or glutathione-mediated S-thiolation [88]. It is a possible mechanism of ALR2 inactivation by TNIC-ThS, which is a NO-donor, NO being an endogenous regulator of ALR2, and the polyol pathway can be useful in preventing secondary diabetic complications.

### 3.5. Acute Toxicity of the TNIC-ThS in Mice

The TNIC-ThS LD50 value was 40 ± 3.37 mg/kg. After the administration of the maximum tolerated dose (30 mg/kg), the following criteria were monitored daily:

- no change in the mice’s condition and no behavioral changes;
- good intensity of motor actions;
- no tonic convulsions;
- normal response to touch, pain, sound, and light stimuli;
- no changes in frequency, depth of respiratory movements, and heart rate;
- skin and hair condition was good;
- no changes in color of mucous membranes and pupil size.

We have demonstrated the antidiabetic activity and acute toxicity of TNIC-ThS. The complex can be recommended for further in vivo study on an experimental model of T2DM.

## 4. Conclusions

Our work has shown that the studied nitrosyl iron complex with thiosulfate ligands (TNIC-ThS) interacts with the PC liposomes membrane, both in the hydrophobic region, close to the glycerol group of lipid molecules, and in the hydrophilic region of the head groups. TNIC-ThS has joint antioxidant, ALR2 inhibitory, and antiglycation activities. TNIC-ThS was found to be an efficient ROS scavenger. TNIC-ThS prevents vesperlysines-like AGEs formation more effectively than aminoguanidine in such an assay. The antiglycation activity of the complex is partially determined by the action of NO released upon hydrolysis. TNIC-ThS was shown to be non-competitive inhibitor of ALR2, probably due to the NO-induced nitrosation of accessible cysteines in the ALR2 active site. Facing these complementary activities, TNIC-ThS can be considered as a good example of a therapeutic agent for the treatment of T2DM and its chronic complications.
